# Mitochondrial Dysfunction as a Context‐Dependent Driver and Amplifier of the SASP: Targeting the mtDNA–cGAS–STING Axis for Next‐Generation Senotherapeutics

**DOI:** 10.1111/jcmm.71419

**Published:** 2026-11-05

**Authors:** Hamza Kamaran Hama, Mohammed Ali Hama, Musa M. Zorab, Amirreza Gholami, Ammar Mohammed Al‐Farga

**Affiliations:** ^1^ Department of Special Education, Sharazur Educational College University of Halabja Halabja Iraq; ^2^ Department of Clinical Biochemistry, College of Science University of Halabja Halabja Iraq; ^3^ Department of Chemistry Dalhousie University Halifax Nova Scotia Canada; ^4^ Department of Food Science and Nutrition, Faculty of Agriculture and Food Ibb University Ibb City Yemen

**Keywords:** cellular senescence, cGAS–STING signalling, mitochondrial DNA release, senescence‐associated secretory phenotype, senomorphic therapeutics

## Abstract

Cellular senescence combines stable cell‐cycle arrest with mitochondrial and innate immune remodelling that shapes the senescence‐associated secretory phenotype (SASP). This narrative review evaluates mitochondrial dysfunction as a context‐dependent initiator or amplifier of senescence and examines mitochondrial DNA (mtDNA) sensing through cGAS–STING as a driver of SASP regulation. We distinguish mitochondrial damage, cytosolic mtDNA exposure, pathway activation, SASP transcription, secretion, paracrine activity and tissue outcomes as separate levels of evidence. The strongest mechanistic studies link BAX/BAK‐mediated minority mitochondrial outer‐membrane permeabilization, VDAC1 oligomerization, defective mitophagy or abnormal mtDNA composition to cytosolic mtDNA, cGAS–STING activation and SASP modulation through genetic or pharmacological perturbation. However, pathway architecture varies across cell types, tissues and senescence triggers, and nuclear DNA, micronuclei, retroelements and mitochondrial RNA can activate overlapping inflammatory programmes. Preclinical evidence therefore supports STING inhibition primarily as a senomorphic, anti‐inflammatory and tissue‐protective strategy rather than a senolytic intervention. Mitophagy enhancement, inhibition of mtDNA release, targeted protein degradation and precision delivery offer complementary approaches, but human efficacy remains unestablished. Clinical translation will require standardized biomarkers, biologically informed patient selection, pharmacodynamic confirmation, tissue‐restricted or reversible modulation and prospective assessment of antiviral, antitumour and long‐term safety. Overall, the mitochondrial dysfunction–mtDNA–cGAS–STING axis is a mechanistically credible but context‐dependent therapeutic framework whose clinical value will depend on validated pathway engagement and appropriately timed clinical intervention.

## 1 Introduction

Cellular senescence has traditionally been defined by stable or irreversible proliferative arrest, but this cell‐cycle‐centred view does not capture the full biological state [1]. Contemporary frameworks recognize that senescent cells can remain viable and functionally active while undergoing extensive secretory, metabolic and stress‐response remodelling [1]. Primary studies support this broader view: Alzheimer's disease patient‐derived senescence‐like neurons displayed metabolic dysfunction together with a pro‐inflammatory phenotype, whereas H_2_O_2_‐induced senescent macrophages showed coordinated suppression of fatty‐acid β‐oxidation and enhancement of lipogenic programmes [2, 3]. Senescent cells are therefore better described as metabolically remodelled in a context‐dependent manner than as uniformly quiescent or hypermetabolic.

A major pathological feature of senescence is the senescence‐associated secretory phenotype (SASP). Depending on cell type and inducing stimulus, senescent cells may increase the transcription and/or secretion of inflammatory cytokines, chemokines, growth‐regulatory factors and tissue‐remodelling mediators [2, 3, 4]. These outputs can exert substantial non‐cell‐autonomous effects. Senescence‐like neurons induced astrogliosis in human astrocytes, conditioned medium from senescent macrophages promoted hepatic stellate‐cell activation and SASP factors from irradiated glioblastoma cells activated JAK–STAT3 and NF‐κB signalling in neighbouring tumour cells [2, 3, 4]. Thus, senescence can remodel surrounding tissue through paracrine mechanisms rather than functioning solely as a cell‐autonomous arrest state.

Intervention studies further support the functional importance of persistent senescent cells. Senolytic removal of senescent myoblasts in myotonic dystrophy type 1 reduced SASP expression and improved proliferative, differentiation and engraftment capacities, whereas ABT263‐mediated clearance of senescent cells accelerated healing of chronic skin wounds in diabetic mice [5, 6]. In irradiated glioblastoma models, elimination of senescent cells through cIAP2 targeting prevented tumour recurrence [4]. These findings demonstrate that senescent cells and their inflammatory outputs can contribute functionally to disease, while also emphasizing substantial tissue and context dependence.

Therapeutically, this biology has led to two related but distinct strategies: senolytics, which eliminate senescent cells and senomorphics, which suppress or remodel pathogenic features such as the SASP [1, 7]. Senolytic studies show that reducing senescent‐cell burden can diminish SASP‐associated effects and improve disease‐relevant outcomes in defined settings [4, 5, 6]. However, senescent‐cell clearance does not necessarily identify or extinguish the proximal pathways generating inflammatory signalling, whereas suppression of selected SASP outputs may leave upstream stress circuitry intact. This distinction creates a mechanistic gap between eliminating senescent cells, reducing their secretory phenotype and defining the damage‐sensing pathways that sustain chronic inflammation.

Although mitochondrial dysfunction, cellular senescence, the SASP and cGAS–STING signalling have each been extensively investigated, their mechanistic intersection remains difficult to interpret because studies measure different biological levels. Mitochondrial dysfunction, cytosolic DNA accumulation, cGAS–STING activation, SASP transcription or intracellular protein expression, SASP secretion, senescence‐associated markers and tissue‐level outcomes are related but mechanistically distinct endpoints. Moreover, cGAS–STING activation during senescence does not by itself establish mitochondrial DNA (mtDNA) as the initiating ligand because cytoplasmic chromatin fragments, micronuclei, retroelement‐associated nucleic‐acid stress and other damage‐associated signals can converge on the same innate immune pathway [8, 9]. Distinguishing both the source of the activating nucleic acid and the mechanistic depth of the evidence is therefore essential for determining when mitochondrial dysfunction can reasonably be positioned upstream of SASP regulation.

Mitochondrial dysfunction provides a plausible bridge between persistent senescence and innate immune activation, although its temporal position within the senescent programme is unlikely to be universal. In astrocytes exposed to combined lead and angiotensin II, cytosolic mtDNA accumulation accompanied cGAS phase separation, whereas inhibition of mtDNA replication reduced cytosolic mtDNA, cGAS condensation, senescence‐associated proteins and SASP expression [10]. Separately, radiation‐induced senescent cancer cells containing micronuclei exhibited cGAS–STING activation and senolytic elimination attenuated both SASP output and DNA‐sensing activity [11]. These observations support mitochondrial danger signalling as an upstream or reinforcing mechanism in selected senescent states but do not establish an invariant mitochondrial dysfunction–mtDNA–cGAS–STING–SASP sequence.

Accordingly, the distinctive contribution of this review is a critical, evidence‐graded and context‐dependent synthesis of the mitochondrial dysfunction–mtDNA–cGAS–STING–SASP axis. Rather than treating mitochondrial dysfunction, cytosolic mtDNA exposure, cGAS–STING activation, SASP expression, secretion and tissue outcomes as interchangeable observations, we evaluate them as separate mechanistic levels of evidence. We further distinguish mitochondrial from competing sources of cytosolic nucleic acids and consider cGAS and STING as mechanistically and therapeutically non‐equivalent targets. Through this framework, we assess whether mitochondrial dysfunction acts as an initiating signal, reinforcing mechanism or downstream consequence across senescent states and clarify the evidence supporting interception of this mitochondrial–innate immune axis as a senomorphic or broader senotherapeutic strategy.

### 1.1 Literature Search Strategy

A targeted literature search was conducted using PubMed, Scopus and Web of Science to identify studies examining the relationships among mitochondrial dysfunction, mtDNA release, cGAS–STING signalling, cellular senescence and the SASP. Search terms included combinations of ‘mitochondrial dysfunction,’ ‘mitochondrial DNA,’ ‘mtDNA release,’ ‘cGAS–STING,’ ‘cellular senescence,’ ‘SASP,’ ‘mitophagy,’ and ‘senotherapeutics.’ Priority was given to mechanistic primary studies, recent preclinical investigations, human studies where available and highly relevant reviews that clarified pathway biology. Studies were evaluated according to the level of evidence linking mitochondrial damage, mtDNA exposure, cGAS–STING activation, SASP regulation and tissue‐level outcomes. As this article is a narrative review, study selection was guided by mechanistic relevance to the scope of the review rather than by formal systematic‐review inclusion criteria.

## 2 Mitochondrial Dysfunction as a Source of Senescence‐Associated Danger Signalling

Mitochondrial dysfunction is linked to cellular senescence through alterations in bioenergetics, redox homeostasis, organelle dynamics and mitochondrial quality control. For the purposes of this review, however, the key question is not whether senescent cells exhibit a uniform mitochondrial phenotype, but which mitochondrial alterations can generate or sustain damage signals capable of reinforcing the SASP. Experimental studies indicate that metabolic disruption, altered fission–fusion dynamics, mitochondrial ROS (mtROS) and defective mitophagy can each contribute to senescence in defined settings, but their relevance to the mitochondrial–innate immune axis depends on whether they promote mitochondrial damage, persistence of dysfunctional organelles or exposure of mtDNA to cytosolic surveillance. CRAT‐deficient human dermal fibroblasts illustrate this mechanistic connection particularly clearly: mitochondrial dysfunction was accompanied by cytosolic mtDNA accumulation, cGAS–STING/NF‐κB activation and increased SASP secretion, while fibroblast‐specific CRAT deletion produced senescence‐associated and inflammatory phenotypes in vivo [12]. Thus, mitochondrial abnormalities should not be considered a universal initiating event in senescence. Depending on cell lineage, stressor and stage of the senescent programme, they may initiate stress, reinforce established senescence, maintain inflammatory signalling or arise downstream of other damage responses. The following sections therefore consider metabolic remodelling, mitochondrial dynamics, mtROS and mitophagy primarily in terms of their ability to generate or preserve the mitochondrial damage state that can feed mtDNA‐dependent innate immune signalling.

### 2.1 Metabolic Remodelling and Mitochondrial Stress

Senescent cells exhibit substantial metabolic remodelling, but the direction of this change is not uniform. SIRT5 depletion in microglial cells increased mitochondrial protein succinylation, impaired mitochondrial adaptability and shifted metabolism toward glycolysis, whereas metabolic dysfunction preceding senescence has also been demonstrated in arsenite‐exposed cardiomyocytes [13, 14]. Replicatively senescent endothelial progenitor cells similarly showed impaired OXPHOS coupling with increased glycolytic activity, whereas DNA‐damage‐induced senescent hepatocytes exhibited greater reliance on OXPHOS [15, 16]. These apparently divergent findings indicate that neither increased glycolysis nor increased mitochondrial respiration constitutes a universal metabolic signature of senescence.

For the mitochondrial–innate immune axis, the mechanistically relevant issue is therefore not the direction of metabolic flux itself but whether metabolic remodelling compromises mitochondrial homeostasis sufficiently to promote oxidative damage, defective organelle turnover, membrane perturbation or mtDNA exposure. Accordingly, metabolic measurements alone should not be considered evidence for activation of the mtDNA–cGAS–STING pathway unless they are linked experimentally to mitochondrial damage and downstream innate immune signalling.

### 2.2 Mitochondrial Dynamics and Membrane Integrity

Altered mitochondrial fission and fusion can modify senescence phenotypes, but morphology alone does not identify a pathogenic mechanism. Excessive fusion has been associated with senescence in FGF21‐deficient mesenchymal stem cells and PGAM5‐deficient retinal pigment epithelial cells, whereas stress‐induced DRP1‐dependent hyperfission promotes senescence in cardiomyocytes and endothelial cells [17, 18, 19]. Conversely, experimentally enhanced fission suppressed SASP‐associated features in some senescent fibroblast models.

These apparently opposing effects indicate that the critical variable is not fragmentation or elongation per se, but whether mitochondrial dynamics preserve bioenergetic integrity and quality control or instead facilitate accumulation of damaged mitochondria. This distinction becomes directly relevant to the present review when mitochondrial remodelling alters membrane permeability or mtDNA localization. For example, DRP1–VDAC1‐dependent mitochondrial remodelling has been linked to mtDNA escape and cGAS–STING activation in stressed endothelial cells. Mitochondrial dynamics should therefore be interpreted as an upstream modifier of mitochondrial integrity rather than as an independent universal hallmark of the senescent state.

### 2.3 Mitochondrial ROS as a Damage‐Amplifying Signal

Mitochondrial reactive oxygen species (mtROS) provide a functional link between mitochondrial dysfunction and the maintenance of senescence‐associated phenotypes. In doxorubicin‐treated human umbilical‐vein endothelial cells, increased mitochondrial superoxide was accompanied by DNA damage, telomeric dysfunction and senescence, whereas the mitochondria‐targeted antioxidant MitoQ reduced mtROS and attenuated senescence‐associated outcomes [20]. Similarly, CRIF1‐deficient endothelial cells accumulated mtROS and senescence markers in association with reduced SIRT3‐dependent antioxidant capacity, while SIRT3 overexpression increased MnSOD, decreased mtROS and reduced senescence‐associated markers [21]. These intervention studies indicate that mitochondrial redox imbalance can contribute functionally to senescence rather than merely accompany it.

Comparable effects have been reported in other cellular contexts. In vascular smooth‐muscle cells, mitochondrial zinc accumulation increased mtROS, activated NF‐κB signalling and promoted senescence, whereas MitoTEMPO reduced both inflammatory signalling and senescence‐associated changes [22]. These findings support mtROS as a context‐dependent amplifier of DNA damage and stress‐responsive inflammatory signalling.

For the mitochondrial–innate immune axis considered here, however, increased mtROS should not be interpreted as direct evidence of mtDNA release or cGAS–STING activation. Its mechanistic relevance lies primarily in its capacity to damage mitochondrial components, impair mitochondrial homeostasis and potentially increase the probability that mtDNA becomes accessible to cytosolic surveillance. Thus, mtROS may act as an initiating signal in some models and as an amplification or maintenance mechanism in others, but its position upstream of the mtDNA–cGAS–STING–SASP cascade requires direct demonstration of mtDNA exposure and downstream pathway activation.

### 2.4 Mitophagy Failure as a Link Between Mitochondrial Damage and mtDNA Sensing

Defective mitochondrial quality control provides a mechanism through which dysfunctional, ROS‐producing mitochondria can persist and reinforce senescence‐associated signalling [23, 24]. In cigarette‐smoke‐exposed airway epithelial cells and experimental COPD models, loss of PRKN impaired mitophagy, increased damaged mitochondrial accumulation, elevated mtROS and intensified cellular senescence, whereas PRKN overexpression restored mitophagy and attenuated these abnormalities [23]. High‐glucose‐treated renal tubular epithelial cells showed reduced PINK1 and OPTN expression, defective mitophagosome formation and increased senescence; OPTN overexpression promoted mitophagy and reduced senescence, whereas inhibition of mitophagy worsened the phenotype [24]. In renal specimens from patients, OPTN expression decreased with greater tubulointerstitial injury and was inversely associated with renal dysfunction, providing human observational support while not independently establishing causality [24]. In oxidative‐stress‐induced and replicatively senescent mesenchymal stem cells, dimethyloxalylglycine improved mitochondrial function and enhanced HIF‐1α/BNIP3‐dependent mitophagy, while BNIP3 knockdown substantially weakened its anti‐senescence effect [25].

Failure of mitochondrial quality control may also connect organelle dysfunction to innate immune signalling. CRAT‐deficient fibroblasts exhibited mitochondrial damage together with cytosolic mtDNA accumulation, cGAS‐STING/NF‐κB activation and increased SASP secretion [12]. In high‐glucose‐treated endothelial cells, DRP1‐VDAC1‐dependent mitochondrial fission promoted mtDNA escape and cGAS‐STING activation; inhibition of DRP1 or STING reduced senescence, whereas pathway activation weakened the protective effect of the tested intervention [26]. In multiple myeloma, PPTC7‐mediated degradation of BNIP3 and NIX suppressed mitophagy, increased cytosolic mtDNA, activated cGAS‐STING signalling and enhanced SASP‐associated responses; restoration of NIX reduced mtDNA leakage, whereas reactivation of cGAS restored inflammatory signalling [27]. These perturbation experiments provide direct support for a mitophagy‐mtDNA‐cGAS‐STING connection in specific cellular contexts.

Collectively, the evidence supports a model in which defective mitochondrial quality control can permit the persistence of dysfunctional mitochondria and, in selected systems, facilitate mtDNA‐dependent innate immune activation. However, the entire sequence from mitochondrial dysfunction through mitophagy failure, mtDNA escape, cGAS‐STING activation and SASP amplification has not been demonstrated as a universal causal pathway across all forms of cellular senescence. The strongest interpretation is therefore that this axis represents a mechanistically validated but context‐dependent route through which mitochondrial dysfunction can reinforce the inflammatory senescent phenotype. The broader relationship between mitochondrial dysfunction, innate immune activation, the SASP, and its biological consequences is summarized in Figure 1.

**FIGURE 1:**
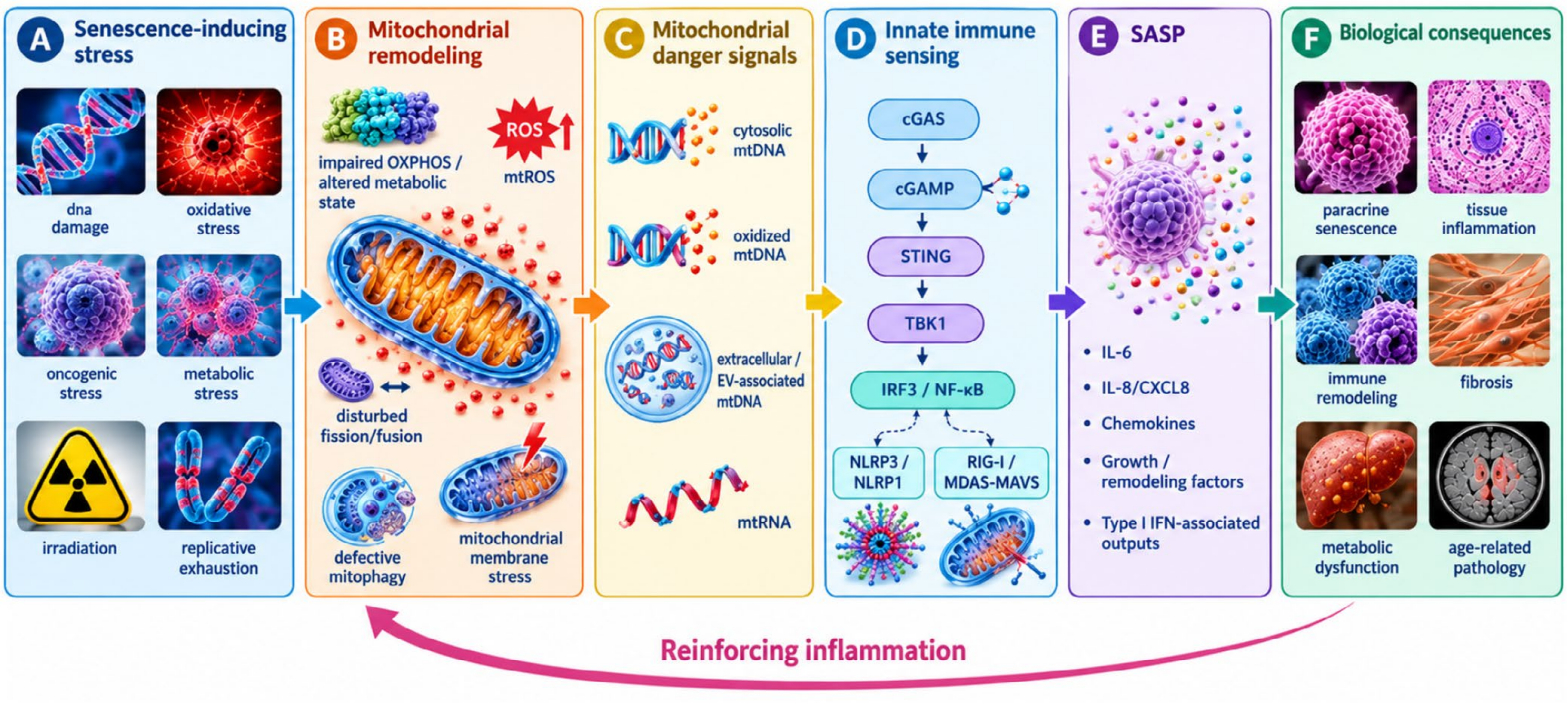
Mitochondrial regulation of the SASP. Senescence‐inducing stresses promote mitochondrial dysfunction and nucleic‐acid release, activating cGAS–STING and related innate immune pathways. The resulting SASP drives inflammation, tissue dysfunction and age‐related pathology through context‐dependent feedback mechanisms.

## 3 The cGAS–STING Innate Immune Axis

Mitochondrial dysfunction becomes immunologically relevant when mitochondrial constituents escape the organelle and enter compartments where they can be detected. Sublethal mitochondrial outer‐membrane permeabilization can release mtDNA into the cytosol, and preventing this release suppresses inflammatory outputs in senescent cells [9]. Increased incorporation of ribonucleotides into mtDNA provides another mechanism: it promotes cytosolic mtDNA release and cGAS–STING activity, producing an mtDNA‐dependent SASP [28]. These observations provide a mechanistic link between mitochondrial dysfunction and innate immunity but do not exclude the possibility that much cGAS–STING activity in senescence is independent of mitochondria.

Interpretation of this literature therefore depends on causal depth. Detection of mitochondrial dysfunction or mtDNA release, cGAS or STING abundance, inflammatory transcription or senescence markers in isolation does not establish the complete sequence: mitochondrial dysfunction → mtDNA release → cGAS → STING → SASP. The strongest evidence comes from systems in which the mitochondrial origin of the DNA signal, cytosolic exposure, pathway activation, downstream SASP regulation and pathway‐directed perturbation are demonstrated within the same experimental framework. Other studies establish only partial links and should be interpreted cautiously. During senescence and aging, cytoplasmic self‐DNA can also arise from chromatin fragments, micronuclei and other nuclear sources that activate cGAS–STING [29, 30]. Mitochondrial damage, cytosolic mtDNA release, cGAS–STING activity and SASP regulation should therefore be treated as distinct experimental readouts rather than interchangeable indicators of a single pathway.

### 3.1 Mechanisms and Source Attribution of Cytosolic mtDNA

Among the physical routes of mtDNA escape described in senescence‐associated systems, minority mitochondrial outer‐membrane permeabilization (miMOMP) provides particularly strong mechanistic evidence. BAX/BAK‐dependent permeabilization in a subset of senescent cells enabled macropore formation and cytosolic mtDNA release; inhibiting mitochondrial outer‐membrane permeabilization reduced inflammatory markers in aged mice [9]. In apoptotic cells, BAX/BAK macropores can expand sufficiently to permit inner‐membrane herniation and release mitochondrial matrix components, including mtDNA [31, 32]. These studies clarify pore mechanics but do not establish BAX/BAK‐mediated mtDNA escape as a universal feature of senescence.

VDAC‐dependent mechanisms provide an additional route for mtDNA release. Under oxidative mitochondrial stress, VDAC oligomerization forms outer‐membrane pores that release mtDNA fragments, whereas pharmacological inhibition with VBIT‐4 reduces mtDNA release and downstream inflammatory signalling [33]. Postmitotic Zmpste24‐deficient myofibers likewise showed mitochondrial dysfunction, impaired mitophagy, VDAC1 oligomerization, mtDNA release, cGAS–STING activation and increased SASP factors; VBIT‐4 reduced mtDNA release, pathway activation and SASP‐associated outputs [34]. In human endothelial cells exposed to high glucose, mitochondrial fission increased DRP1 translocation and its interaction with VDAC1, promoting VDAC1 oligomerization, cytosolic mtDNA escape, cGAS–STING signalling and senescence. Pathway‐directed interventions substantially attenuated these effects [26]. By contrast, loss of ERβ in colonic epithelium caused mitochondrial fission, mtDNA leakage and cGAS–STING activation without identifying a specific BAX/BAK‐ or VDAC‐dependent pore‐forming step [35]. These findings support multiple routes of mtDNA escape rather than a single dominant mechanism.

Defective mitochondrial and nucleic‐acid quality control can expose mtDNA without a defined membrane pore. In senescent and age‐associated inflammatory models, ribonucleotide‐loaded mtDNA was released into the cytosol, whereas deoxyribonucleoside supplementation inhibited the mtDNA‐dependent SASP [28]. Restoring lysosomal mtDNA processing in aged haematopoietic stem cells reduced cGAS–STING signalling and inflammatory programmes and improved repopulating function [36]. This finding identifies an aging‐related quality‐control mechanism but does not establish that the haematopoietic stem cells were senescent. Thus, BAX/BAK‐mediated permeabilization, VDAC oligomerization, mitochondrial remodelling and defective mtDNA quality control represent distinct but complementary mechanisms that permit mtDNA to reach cytosolic sensors.

Because cGAS detects DNA without identifying its origin, source attribution is essential. Damaged cytoplasmic chromatin fragments of nuclear origin can activate cGAS–STING and induce the SASP in primary senescent cells and in vivo [29], whereas genetic studies have identified inflammatory transcriptional programmes driven by cGAS‐dependent sensing of cytoplasmic DNA without establishing a mitochondrial source [30]. Micronuclear DNA generated by genomic instability is another potential nuclear source of cGAS activation, particularly in therapy‐associated settings. Age‐associated LINE‐1 activation has also been implicated in cGAS–STING signalling in naturally aged myocardium, showing that non‐mitochondrial nucleic‐acid stress can converge on the same downstream pathway [37]. A stressed or senescent cell may therefore contain several non‐mutually exclusive sources of activating nucleic acids, including mitochondrial dysfunction, chromatin instability, micronuclei and derepressed retroelements.

Evidence should qualify as direct support for an mtDNA–cGAS–STING mechanism only when the mitochondrial origin of the activating DNA is demonstrated directly or implicated functionally by manipulating mtDNA abundance, integrity, localization or release. Total cellular mtDNA, damaged mtDNA, cytosolic mtDNA and extracellular mtDNA are therefore not interchangeable endpoints. Distinct experiments have tracked oxidatively damaged mtDNA released from mitochondria using fluorescently labelled wild‐type and mutant mtDNA constructs and identified mtDNA within extracellular vesicles exported by senescent cells. These approaches describe related but different processes of mitochondrial nucleic‐acid transport [34, 38].

### 3.2 Intracellular Sensing and STING Activation

Direct pathway mapping is strongest when measurements of mtDNA availability are coupled to downstream cGAS–STING activity. In endothelial cells exposed to oscillatory shear stress, mitochondrial damage increased cytosolic mtDNA, cGAS‐dependent cGAMP production and STING activation; endothelial STING deficiency or pharmacological inhibition alleviated senescence‐associated and atherosclerotic phenotypes [39]. In inflamed odontoblast‐like cells, cytosolic mtDNA generated by LPS‐induced mitochondrial injury contributed to inflammatory responses. mtDNA depletion suppressed cGAS–STING signalling and nuclear translocation of p65 and IRF3, whereas mtDNA repletion restored these events in a STING‐dependent manner [40]. In rotenone‐treated THP‐1‐derived macrophages, STING and TBK1 phosphorylation increased, while STING inhibition attenuated NF‐κB activation, NLRP3 expression and IL‐1β release [41]. These studies also illustrate the need to distinguish pathway abundance from pathway activity. Increased cGAS or STING expression alone does not establish activation. Evidence is stronger when studies demonstrate cGAMP production, STING or TBK1 phosphorylation, pathway trafficking, downstream transcription‐factor activation or functional dependence on genetic or pharmacological perturbation.

Canonical cGAS–STING activation is not universal in senescence. In serially passaged, senescent and progeroid human fibroblasts, cGAS‐ and STING‐dependent inflammatory programmes occurred without increased cGAMP production, with reduced STING phosphorylation and without conventional perinuclear STING trafficking. Instead, a noncanonical STING state was detected at the endoplasmic reticulum, nuclear envelope and chromatin [42]. Thus, the canonical mtDNA–cGAS–cGAMP–phospho‐STING–TBK1–IRF3 pathway is well documented in relevant inflammatory systems but only incompletely and variably documented in senescence. The principal mechanisms of mtDNA release and canonical or context‐dependent cGAS–STING activation are summarized in Figure 2.

**FIGURE 2:**
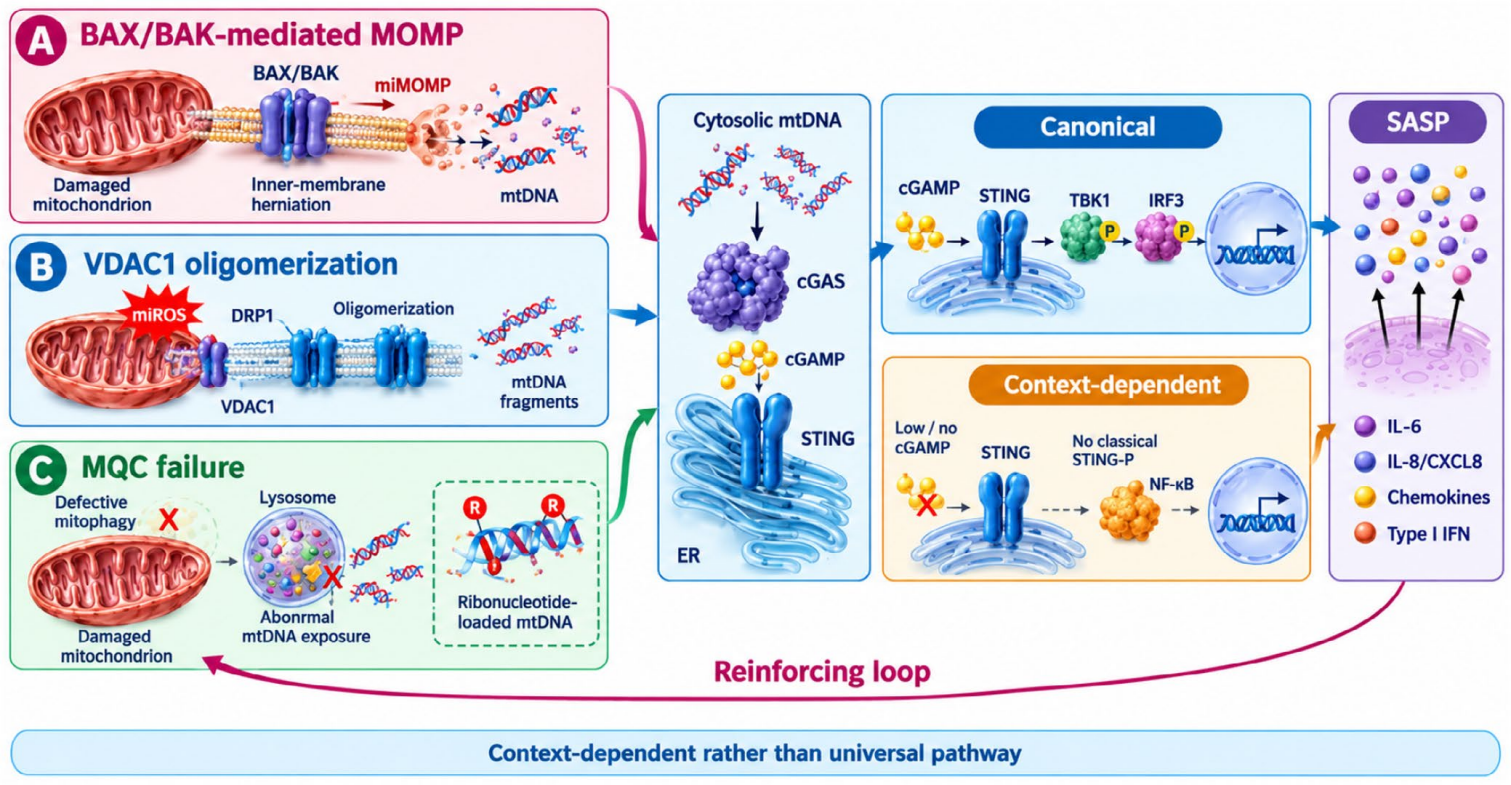
Mechanisms linking mitochondrial damage to SASP regulation. BAX/BAK‐mediated permeabilization, VDAC1 oligomerization and mitochondrial quality‐control failure promote mtDNA release and context‐dependent cGAS–STING signalling, leading to SASP production and reinforcing inflammation.

Heterogeneity also has therapeutic implications because cGAS and STING are not biologically equivalent targets. cGAS is classically defined as a DNA sensor that produces 2′3′‐cGAMP upstream of STING, whereas STING functions as a downstream signalling hub; however, both proteins have functions beyond this linear pathway. Whole‐body cGAS deficiency during natural aging was associated with multi‐organ inflammation, increased frailty and reduced median lifespan. A nuclear role for cGAS in heterochromatin maintenance was implicated independently of its established cytosolic DNA‐sensing functions [43]. Lifelong genetic loss of cGAS therefore does not model selective pharmacological inhibition of mtDNA sensing in an aged individual.

STING‐directed interventions show a distinct preclinical pattern. Genetic deletion or pharmacological inhibition of STING attenuates inflammatory or disease‐associated phenotypes in models of vascular, neurological, metabolic and age‐related pathology. These findings indicate context‐specific benefit rather than universal pathogenicity. STING also supports antiviral defence, immune surveillance and context‐dependent antitumour immunity. cGAS and STING should therefore be treated as distinct therapeutic nodes within a broader signalling network. The effects of targeting either molecule will depend on subcellular function, tissue context, reversibility, signal duration and the extent to which protective innate immune functions are preserved.

### 3.3 Transcriptional Activation of the SASP

Genetic perturbation provides causal evidence that cGAS–STING regulates the SASP in several senescence models. cGAS deletion abrogated SASP responses induced by spontaneous immortalization and by radiation‐ or etoposide‐induced senescence in mouse embryonic fibroblasts, while STING‐dependent sensing of cytoplasmic chromatin promoted the SASP in primary human cells and in vivo [29, 30]. Evidence implicating mtDNA further defines this relationship. miMOMP‐dependent cytosolic mtDNA activated cGAS–STING and modulated the SASP, whereas ribonucleotide‐driven mtDNA release generated a mitophagy‐independent SASP that was reduced by deoxyribonucleoside supplementation [9, 28]. These interventions establish the functional relevance of mtDNA availability rather than a merely correlative association.

However, downstream transcriptional responses vary by model. CRAT knockdown induced mitochondrial dysfunction, cytosolic mtDNA accumulation, cGAS–STING/NF‐κB activation and SASP secretion in human dermal fibroblasts; fibroblast‐specific CRAT loss also increased SASP levels and skin inflammation in mice [12]. In metabolically stressed pancreatic β‐cells, cGAS or STING silencing and pharmacological STING inhibition suppressed SASP output and senescence‐associated phenotypes, whereas mtDNA depletion reversed pathway activation [44]. These perturbation experiments support a functional mtDNA‐sensing pathway but do not imply that its downstream architecture is identical across senescent states.

The non‐cell‐autonomous nature of this signalling expands the potential impact of mitochondrial nucleic‐acid release. Therapy‐induced senescent tumour cells released extracellular vesicles containing mtDNA; transfer to PMN‐MDSCs activated cGAS–STING–NF‐κB signalling and enhanced immunosuppressive function, representing a non‐cell‐autonomous effect rather than a cell‐autonomous SASP branch [38]. Likewise, a Salmonella genotoxin caused mtDNA damage and cytosolic release, driving cGAS–STING‐dependent type I interferon signalling and the production of inflammatory SASP factors that promoted T‐cell senescence [45]. Mitochondrial nucleic‐acid signalling can therefore alter both the senescent cell and nearby immune or stromal populations.

SASP endpoints must also be distinguished. Evidence may measure SASP transcription, intracellular protein expression, secretion or functional paracrine activity. SASP secretion was demonstrated in CRAT‐deficient fibroblasts, whereas other studies measured only transcript or intracellular protein levels of selected SASP factors [12, 34]. These endpoints should not be treated as interchangeable measures of secretory output.

Collectively, these studies demonstrate different levels of causal support rather than a single pathway architecture. The strongest senescence‐specific evidence links mitochondrial mtDNA availability or release to cGAS–STING activation and SASP regulation through pathway‐directed perturbation. Other models connect only parts of this sequence, leaving the relationships among mitochondrial stress, DNA sensing, inflammatory transcription and specific senescence‐associated phenotypes less certain. cGAS–STING therefore appears to act more often as a context‐dependent regulator of SASP development and persistence than as an obligatory driver of senescent arrest. Claims about the mitochondrial dysfunction–mtDNA–cGAS–STING–SASP axis should be limited to systems in which mitochondrial origin, cytosolic mtDNA exposure, pathway activation and downstream SASP responses are functionally linked. Table 1 summarizes the mechanistic depth and principal limitations of representative studies.

**TABLE 1:** Mechanistic evidence supporting the mitochondrial dysfunction–mtDNA–cGAS–STING–SASP axis.

Study	Experimental model	Mechanistic evidence	Main intervention	Causal depth	Key limitation
[9]	Senescent cells and aged mice	miMOMP → mtDNA release → cGAS–STING → SASP	BAX/BAK–MOMP suppression	Very high	Generalizability across senescence models is uncertain
[28]	Senescent and inflammatory‐aging models	Ribonucleotide‐loaded mtDNA → cGAS–STING → SASP	Deoxyribonucleoside supplementation	Very high	Relevance across different senescence triggers remains unclear
[12]	CRAT‐deficient fibroblasts and mice	Mitochondrial dysfunction → cytosolic mtDNA → cGAS–STING/NF‐κB → SASP secretion	CRAT perturbation	High	mtDNA‐release mechanism was not identified
[26]	High‐glucose‐treated endothelial cells	DRP1–VDAC1 fission → mtDNA escape → cGAS–STING → senescence	DRP1/STING inhibition	Moderate–high	SASP secretion was not directly established
[44]	Metabolically stressed pancreatic β cells	Cytosolic mtDNA → cGAS–STING → SASP and senescence	mtDNA depletion; cGAS/STING inhibition	High	mtDNA‐release route remains unresolved
[39]	Shear‐stressed endothelial cells	Mitochondrial damage → mtDNA → cGAMP/STING → vascular senescence	STING deletion/inhibition	Moderate–high	Limited direct SASP characterization
[40]	LPS‐treated odontoblast‐like cells	mtDNA → cGAS–STING → p65/IRF3 signalling	mtDNA depletion/repletion	Moderate	Inflammatory rather than senescence‐specific model
[41]	Rotenone‐treated macrophages	Oxidized mtDNA → STING/TBK1 → NF‐κB/NLRP3	STING inhibition	Moderate	Complete senescence–SASP axis was not tested

*Note:* Causal depth was qualitatively assigned according to how completely each study experimentally connected mitochondrial damage or mtDNA release, cGAS–STING activation, downstream SASP or senescence‐associated responses and pathway‐directed perturbation or rescue. Very high indicates experimental support across nearly the complete mechanistic sequence together with pathway‐directed intervention; high indicates strong causal evidence with one important mechanistic step remaining unresolved; moderate–high indicates functional linkage of several pathway components with intervention evidence but incomplete characterization of the full mtDNA–cGAS–STING–SASP sequence; and moderate indicates partial mechanistic linkage or evidence derived from models that do not directly establish the complete senescence–SASP axis. These categories are intended as a qualitative assessment of mechanistic completeness rather than a formal risk‐of‐bias or evidence‐certainty grading system.

Abbreviations: miMOMP, minority mitochondrial outer‐membrane permeabilization; mtDNA, mitochondrial DNA; SASP, senescence‐associated secretory phenotype.

### 3.4 Tissue‐Specific and Immune Responses

Marked tissue heterogeneity makes it difficult to define a common mitochondrial–cGAS–STING senescence programme. In lung fibroblasts from patients with idiopathic pulmonary fibrosis, cytosolic and extracellular mtDNA were elevated. cGAS inhibition, cGAS knockdown or DNase treatment reduced senescence‐associated measures, including IL‐6, whereas ectopic mtDNA exacerbated senescence in a cGAS‐dependent manner [46]. In lung tissue from patients with idiopathic pulmonary fibrosis, primary alveolar epithelial cells similarly exhibited increased cytosolic and supernatant mtDNA; cGAS inhibition or knockdown reduced senescence‐associated responses [47]. STING deficiency attenuated oscillatory‐shear‐induced senescence in endothelial models, and cytosolic mtDNA–cGAS–STING signalling was linked to islet inflammation and impaired glucose regulation in metabolically stressed β‐cells [39, 44]. By contrast, colonic epithelial ERβ deficiency and postmitotic muscle aging were associated with mtDNA leakage and cGAS–STING activation but arose from different upstream mitochondrial disturbances [34, 35].

Immune‐cell responses are similarly heterogeneous. In aged microglia, mtDNA in the cytosol activated cGAS–STING, producing reactive transcriptional states, bystander inflammation, neurotoxicity and cognitive impairment in mouse models [48]. In telomerase‐deficient (mTerc−/−) mice with dysfunctional telomeres, senescence‐associated macrophages exhibited mitochondrial dysfunction and aberrant STING and NLRP3 activation. Both STING inhibition and senolytic treatment improved mitochondrial integrity and reduced the acute inflammatory pneumonia associated with severe viral infection [49]. Conversely, senescent human monocyte‐derived macrophages cultured ex vivo showed reduced STING expression and impaired 2′3′‐cGAMP signalling. Monocytes from individuals with obesity likewise exhibited senescence‐associated features, blunted downstream STING signalling and reduced cytokine production [50].

These apparently contradictory observations are biologically informative. Senescence does not preserve uniform DNA‐sensor competence across cell types; chronic inflammation may therefore persist even when acute STING signalling is diminished. Non‐cell‐autonomous effects add further complexity: tumour‐cell‐derived mtDNA can enhance PMN‐MDSC‐mediated immunosuppression, whereas genotoxin‐induced SASP factors can propagate senescence to T cells [38, 45]. These outcomes depend on the form and source of nucleic‐acid stress, sensor competence, cell lineage, disease context and whether signalling is cell autonomous. Figure 3 summarizes the tissue‐specific effects and variable evidence supporting cGAS–STING involvement.

**FIGURE 3:**
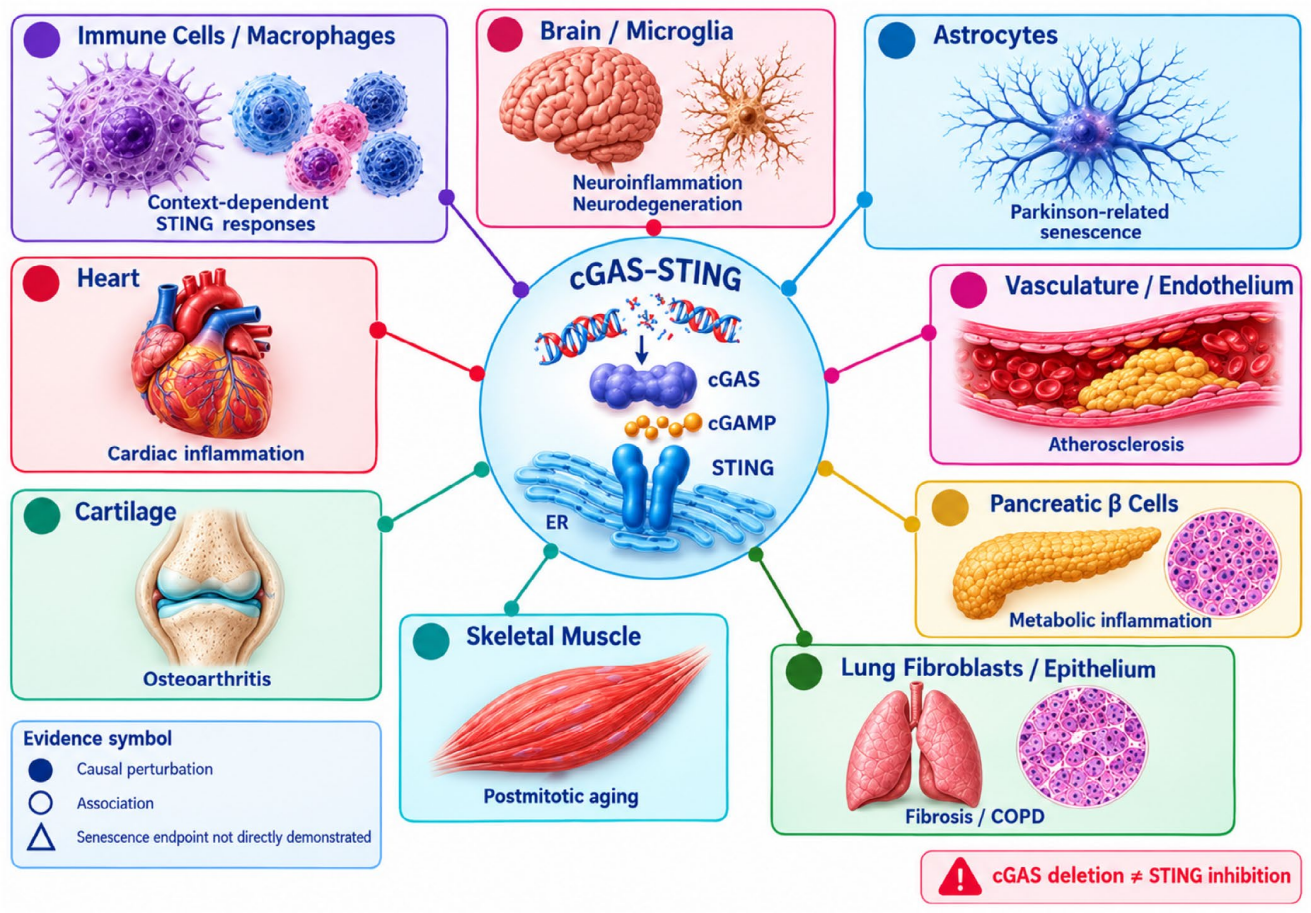
Tissue‐specific cGAS–STING signalling in aging and age‐related diseases. Depending on cellular context, pathway dysregulation contributes to senescence, inflammation, fibrosis, metabolic dysfunction, atherosclerosis and neurodegeneration. Symbols indicate evidence type; cGAS deletion is not equivalent to STING inhibition.

### 3.5 Innate Sensor Crosstalk

Mitochondrial injury can activate innate immune pathways beyond cGAS–STING, arguing against a universal signalling hierarchy. In an immunosenescence model, telomere‐dysfunctional macrophages with mitochondrial stress exhibited aberrant STING and NLRP3 activation [49]. In rotenone‐treated THP‐1‐derived macrophages, oxidized mtDNA accumulated in the cytosol and increased STING/TBK1 phosphorylation and NF‐κB activation. STING inhibition reduced NLRP3 expression and IL‐1β release, supporting a cGAS–STING–NF‐κB axis that contributes to NLRP3 priming in this specific cellular context [41]. This association should not be generalized to all forms of senescence or all modes of inflammasome activation.

NLRP1 provides another example of innate‐sensor crosstalk. Through gasdermin D, NLRP1 promoted senescence markers and SASP‐factor release in mouse and fibroblast senescence models; some studies reported that cGAS sensing of genomic damage contributed to NLRP1 activation [51]. Mitochondrial nucleic‐acid sensing also extends beyond DNA. Cytosolic mitochondrial RNA accumulated in senescent cells and activated RIG‐I and MDA5–MAVS signalling to regulate the SASP. Inhibition of these RNA sensors reduced SASP factors, while BAX/BAK deletion limited mtRNA release and SASP expression in vitro and in vivo [52].

Together, these findings support a distributed innate‐sensing network rather than an isolated cGAS–STING module. The dominant inflammatory route may vary with the released nucleic acid species, membrane escape mechanism, cell lineage and senescence‐inducing stress because cGAS–STING can interact with inflammasome and RNA‐sensing pathways. Mitochondrial signalling should therefore be viewed as one component of the innate immune network regulating the SASP, with cGAS–STING acting as a context‐dependent rather than universally dominant pathway.

## 4 Preclinical Evidence: In Vivo Models of cGAS‐STING in Aging

Mechanistic studies establish that mtDNA release can engage cGAS‐STING and reinforce the SASP, but organism‐level validation requires more than pathway activation: manipulation of the axis must alter age‐associated inflammation, senescence burden, tissue dysfunction or disease progression. In physiologically aged mice, retinal type I interferon upregulation correlated with cytosolic mtDNA accumulation and cGAS/STING activation; pharmacological induction of mitophagy with urolithin A attenuated pathway activation and ameliorated deterioration of neurological function [53]. In a separate aged‐mouse study, inhibition of minority mitochondrial outer membrane permeabilization reduced inflammatory markers and improved healthspan, consistent with suppressing an upstream source of mtDNA‐driven cGAS‐STING/SASP signalling [9]. These interventions support a functional link between mitochondrial stress and inflammatory aging, but they do not establish that direct, lifelong loss of cGAS‐STING is uniformly beneficial. Indeed, cGAS‐knockout mice displayed accelerated aging, multi‐organ inflammation, increased frailty and shortened median lifespan, with evidence that nuclear cGAS maintains heterochromatin organization independently of its cytosolic DNA‐sensing role [54]. Current animal evidence therefore moves the pathway beyond association toward experimental actionability, while simultaneously arguing against treating cGAS‐STING as a single, uniformly deleterious aging switch.

### 4.1 Insights From STING Knockout Models

The strongest causal evidence comes from genetic perturbation in disease‐relevant animals, although most direct knockout studies test age‐related pathology rather than physiological aging itself. In 5×FAD mice, cGAS deletion protected against cognitive impairment, amyloid‐β pathology and neuroinflammation, while the STING inhibitor H‐151 suppressed pathway activation and ameliorated Alzheimer‐related pathogenesis in the same model [55]. In a mouse model of Parkinson's disease, astrocyte‐specific cGAS deletion prevented astrocyte senescence and neurodegeneration [56], providing cell‐type‐restricted evidence that cGAS can causally support a senescence‐linked neural phenotype rather than merely accompany inflamed tissue. Vascular models provide similarly direct evidence for STING. In Apoe−/− mice, Sting deletion reduced atherosclerotic lesions, plaque lipid and macrophage accumulation and aortic inflammatory molecule expression; C‐176 also ameliorated atherogenesis, whereas bone‐marrow‐specific STING expression promoted it [57]. Independently, Apoe−/−Sting1−/− mice showed reduced vascular damage and atherosclerosis burden with lower type I interferon production [58]. These convergent loss‐, gain‐ and pharmacological‐intervention data give STING substantial causal weight in chronic vascular inflammation. However, the opposite whole‐organism phenotype of cGAS deficiency during natural aging is a major constraint on generalization: cGAS‐knockout mice developed greater inflammation, frailty and reduced median lifespan through a reported nuclear chromatin‐maintenance function distinct from catalytic DNA sensing [54].

Taken together, the knockout evidence argues against treating cGAS and STING as therapeutically equivalent components of a single linear pathway. STING loss or inhibition has shown reproducible protection in several models of chronic inflammatory pathology, whereas lifelong cGAS deficiency can produce detrimental aging phenotypes through functions that extend beyond canonical cytosolic DNA sensing. Genetic deletion of cGAS therefore cannot be assumed to phenocopy selective STING inhibition, and neither intervention necessarily predicts the effects of temporally controlled pharmacological treatment in established disease. At present, the preclinical evidence supports STING more consistently as a tractable downstream amplifier of chronic inflammatory signalling, whereas the therapeutic consequences of cGAS inhibition are more complex because catalytic DNA sensing and noncanonical nuclear functions may have different biological effects.

### 4.2 Age‐Related Pathologies

Neurodegenerative models show convergence across genetic and drug approaches while also clarifying endpoint specificity. In MPTP parkinsonian mice, C‐176 suppressed downstream STING signalling, neuroinflammation, dopaminergic neurotoxicity, motor deficit and NLRP3 activation in vivo [59]. This demonstrates functional neuroprotection, but the study did not report direct reduction of cellular senescence; its evidence therefore supports anti‐inflammatory and tissue‐protective activity rather than senolysis. By contrast, astrocyte‐specific cGAS deletion in Parkinson's disease reduced both astrocyte senescence and neurodegeneration [56], while cGAS deletion and H‐151 treatment in 5×FAD mice improved cognitive, inflammatory and amyloid‐related outcomes [55]. Across these neural models, cGAS‐STING can couple inflammatory glial responses to tissue injury, but the measured contribution of senescence remains model dependent.

Atherosclerosis provides particularly strong intervention evidence. Genetic loss‐of‐function, pharmacological blockade and bone‐marrow‐specific gain‐of‐function showed that STING modulates lesion formation and vascular inflammation in Apoe−/− mice [57]. Independently, reduced vascular damage and plaque burden were observed in Apoe−/−Sting1−/− mice [58]. Neither study established selective clearance of senescent cells. These findings therefore support interruption of inflammatory vascular damage rather than a senolytic mechanism.

The osteoarthritis literature similarly separates structural, inflammatory and senescence endpoints. In a mechanical‐stress model, chondrocyte‐lineage Piezo1 deletion reduced cGAS‐STING activation and cartilage degradation, while STING inhibitors decreased inflammation and slowed osteoarthritis progression [60]. Those outcomes establish pathway contribution to inflammatory cartilage damage but do not by themselves demonstrate reduced senescence. A destabilization‐of‐the‐medial‐meniscus rat study provided a tighter senescence link: LRRK2 overexpression increased cGAS‐STING signalling, chondrocyte senescence, mitochondrial dysfunction and cartilage degeneration, whereas H‐151 partially mitigated the LRRK2‐induced increases in chondrocyte senescence and mitochondrial impairment [61]. Here, STING inhibition is better described as senomorphic or pathway‐suppressive because reduced senescence‐associated phenotypes were reported without evidence of selective senescent‐cell elimination.

Natural aging models extend this distinction beyond induced disease. In naturally aged mice, H‐151 improved cardiac function and reduced cardiac inflammation and senescence phenotypes in a study linking age‐related LINE‐1 activation to cGAS‐STING signalling [62]. In naturally aged one‐year‐old female mice, abnormal uterine receptivity occurred with cytoplasmic DNA accumulation and cGAS‐STING activation and the uterine abnormality was partially rescued by a STING inhibitor [63]. These studies provide direct evidence that suppressing STING‐associated signalling can improve established age‐related functional phenotypes in more than one tissue, but neither demonstrates selective elimination of senescent cells. Because much of the causal literature relies on induced disease models and many pharmacological studies report inflammation or structural protection without cell‐clearance endpoints, the evidence is stronger for modulation of inflammatory phenotypes than for broad organism‐wide geroprotection. Taken together, these animal studies support cGAS‐STING as a recurrent yet tissue‐conditioned inflammatory circuit connecting cytosolic DNA stress to dysfunction and disease progression. The most defensible translational interpretation is that STING inhibition currently has stronger preclinical support as a senomorphic and tissue‐protective strategy than as a next‐generation senolytic; whether this can be achieved safely without disrupting context‐specific protective functions of cGAS remains unresolved. Representative in vivo evidence, therapeutic interpretations and major limitations are summarized in Table 2.

**TABLE 2:** Preclinical evidence for targeting the cGAS–STING axis in aging and age‐related diseases.

Study and model	Intervention	Main outcome	Interpretation	Key limitation
[53]; naturally aged mice	Urolithin A–induced mitophagy	Reduced cytosolic mtDNA–cGAS/STING signalling and improved neurological function	Upstream, tissue‐protective	Direct senescence reduction was not established
[9]; aged mice	miMOMP suppression	Reduced inflammation and improved healthspan	Upstream senomorphic	Not a direct STING‐targeting intervention
[55]; 5×FAD mice	cGAS deletion or H‐151	Improved cognition, amyloid pathology and neuroinflammation	Neuroprotective	Senescence contribution was not fully defined
[56]; Parkinson's model	Astrocyte‐specific cGAS deletion	Reduced astrocyte senescence and neurodegeneration	Senescence‐modifying	Disease‐specific genetic model
[59]; MPTP Parkinson's model	C‐176	Reduced neuroinflammation, neurotoxicity and motor impairment	Anti‐inflammatory	Cellular senescence was not directly assessed
[58]; Pham et al. [57]; Apoe^−^/^−^ mice	STING deletion or C‐176	Reduced vascular inflammation and atherosclerosis	Vascular protective	Senescent‐cell clearance was not demonstrated
[60, 61]; osteoarthritis models	Piezo1 deletion or STING inhibition	Reduced inflammation, cartilage damage and senescence‐associated phenotypes	Senomorphic	No evidence of selective senolysis
[62]; naturally aged mice	H‐151	Improved cardiac function and reduced inflammation and senescence markers	Senomorphic	LINE‐1, rather than mtDNA, was the upstream signal
[63]; aged female mice	STING inhibitor	Partially restored uterine receptivity	Tissue protective	DNA source and senescence contribution remain unclear
[54]; naturally aging mice	Lifelong cGAS deletion	Increased inflammation, frailty and premature aging	Important safety warning	Whole‐body genetic deletion may not predict drug effects

*Note:* Current animal evidence supports STING inhibition primarily as a senomorphic, anti‐inflammatory and tissue‐protective strategy, rather than as a senolytic intervention.

## 5 Limitations of Conventional Senotherapeutics: Rationale for Pathway‐Directed Intervention

Classical senotherapeutic strategies show that altering senescent‐cell burden or phenotype can improve disease‐related outcomes, but they also reveal limitations that motivate pathway‐directed approaches. Senolytics exploit survival mechanisms that allow senescent cells to resist apoptosis. For example, navitoclax and ABT‐263 inhibit BCL‐2‐family proteins, preferentially eliminate senescent populations and improve function in selected experimental models [64, 65]. However, senolytic susceptibility is strongly cell‐type dependent and selectivity is limited when nonsenescent cells require the same survival pathways. BCL‐xL inhibition illustrates this problem because platelet dependence on BCL‐xL causes dose‐limiting thrombocytopenia. Targeted delivery and PROTAC‐based approaches may reduce this liability but do not establish universal senescent‐cell selectivity [66, 67].

Senescent‐cell clearance also does not reliably predict tissue repair. ABT‐263 rejuvenated haematopoietic and muscle stem‐cell compartments in aged or irradiated mice, whereas treatment of advanced atherosclerosis reduced measures of plaque stability and was associated with increased mortality [64, 68]. These divergent outcomes reinforce the distinction among reducing senescent‐cell abundance, suppressing SASP output, restoring tissue architecture and improving organ function.

Phytochemical senotherapeutics introduce a different set of limitations. Quercetin‐containing approaches exhibit context‐ and dose‐dependent biological effects and are constrained by formulation, bioavailability and tissue distribution. Consistent with this heterogeneity, dasatinib plus quercetin has produced benefits in selected experimental settings but did not improve the primary bone‐resorption endpoint in the overall population of a phase 2 study in postmenopausal women, although exploratory responses varied with baseline senescent‐cell burden [69]. These observations do not demonstrate that therapeutic failure results from persistent mtDNA–cGAS–STING signalling; rather, they show that conventional senotherapeutics are limited by variable cellular susceptibility, imperfect selectivity, exposure and incomplete correspondence between cell clearance and tissue recovery.

These limitations provide the rationale for considering mechanistically defined senomorphic strategies. Instead of eliminating the entire senescent cell, pathway‐directed interventions may suppress specific inflammatory circuits that sustain pathological SASP activity. The mitochondrial dysfunction–mtDNA–cGAS–STING axis is of particular interest because it links an upstream damage source to downstream innate immune signalling. However, pathway specificity should not be conflated with senescent‐cell specificity. Targeting this axis is therefore best considered a complementary strategy whose potential advantage lies in mechanistic control of inflammatory signalling rather than an established superiority over senolytic clearance.

## 6 Next‐Generation Senotherapeutics: Pathway‐Directed Targeting of the Mitochondrial–Innate Immune Axis

The therapeutic rationale for targeting the mitochondrial–cGAS–STING axis should be distinguished from classical senolysis. Senolytics eliminate senescent cells through selective induction of cell death, whereas senomorphics or senostatics suppress pathological features of the senescent state without necessarily reducing senescent‐cell abundance. Most interventions directed against cGAS–STING belong to the latter category because they primarily attenuate inflammatory signalling and SASP‐associated outputs rather than selectively eliminating senescent cells. Consequently, targeting this pathway is more appropriately considered a precision senotherapeutic strategy that may complement, rather than replace, conventional senolysis [44, 62, 70]. In primary bronchial epithelial cultures from patients with chronic obstructive pulmonary disease, C‐176 likewise attenuated SASP production and senescence‐marker expression, providing human ex vivo evidence of pathway tractability without demonstrating clinical efficacy [71]. A complementary strategy is to act further upstream: restoring mitochondrial quality control can reduce the damaged‐mitochondrial substrate that feeds cytosolic mtDNA sensing, and direct linkage between mitophagy, cytosolic mtDNA and cGAS‐STING has been demonstrated in aging models [53, 72]. These findings support a mechanistic transition from indiscriminate cell elimination toward interception of the mitochondrial damage‐mtDNA‐cGAS‐STING‐SASP axis, but mechanistic precision is not equivalent to cellular selectivity. The translational challenge is therefore to suppress chronic senescence‐associated innate immune activity sufficiently to reduce tissue inflammation without unnecessarily compromising mitochondrial quality control, tissue repair, host defence or immune surveillance. The principal therapeutic strategies targeting different levels of the mitochondrial–cGAS–STING–SASP axis are summarized in Figure 4.

**FIGURE 4:**
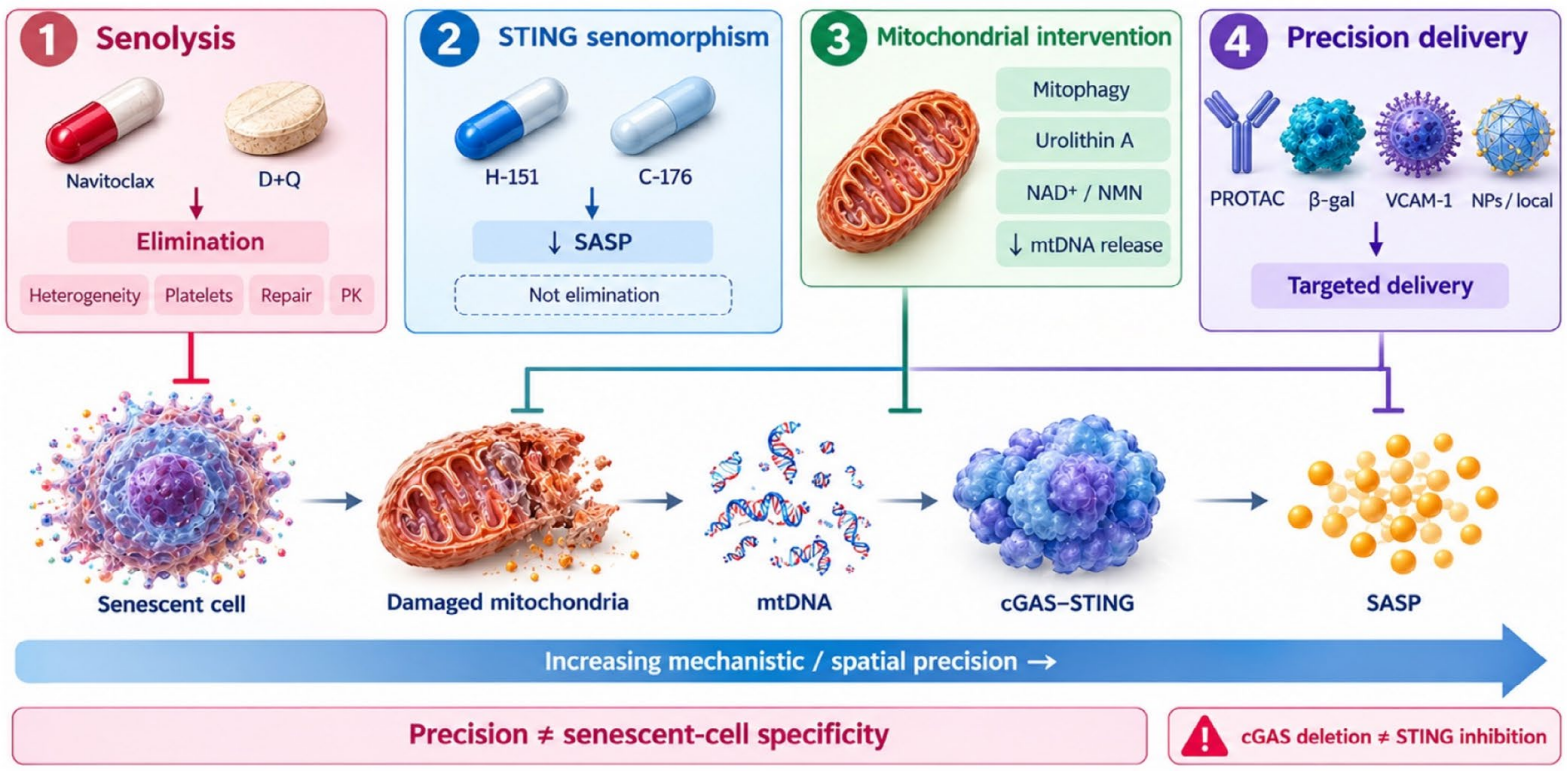
Therapeutic strategies targeting the mitochondrial–cGAS–STING–SASP axis. Senolysis eliminates senescent cells, whereas STING inhibition, mitochondrial restoration and precision delivery suppress specific pathway components without necessarily causing cell clearance. Mechanistic precision does not guarantee senescent‐cell specificity.

### 6.1 Senomorphics: STING Inhibitors Versus PROTACs

Small‐molecule STING inhibitors currently have the broadest experimental support. H‐151 reduced senescence in high‐glucose‐exposed macrophages in a diabetic vascular‐aging model and suppressed cGAS–STING activation and senescence in a LINE‐1‐driven cardiac‐aging model; the latter study also reported improved cardiac function and reduced inflammatory and senescence phenotypes in naturally aged mice [62, 70]. C‐176 reduced SASP‐associated inflammation and senescence in metabolically stressed pancreatic β‐cells and COPD bronchial epithelium, although the COPD evidence came from patient‐derived cultures rather than an interventional trial [44, 71]. In aged mice, three weeks of C‐176 reduced inflammatory cytokines and p21 and restored several mitochondrial and metabolic proteins that had declined with age. These findings indicate that STING suppression can alter a broader mitochondrial–inflammatory state without establishing direct enhancement of mitophagic flux [73]. H‐151 also partially mitigated LRRK2‐associated chondrocyte senescence and mitochondrial impairment in osteoarthritis models [61]. Overall, these studies demonstrate preclinical and ex vivo pathway modulation across several tissues, but none shows that systemic STING inhibition distinguishes senescent from nonsenescent cells.

Targeted degradation represents a mechanistically different proposition because it seeks to reduce STING protein abundance rather than merely inhibit signalling by target occupancy. Among the available studies, the clearest STING‐degradation example is an inhalable, enzyme‐responsive pulmonary nanococktail that released a STING‐degrading PROTAC in a senescence‐associated β‐galactosidase‐responsive compartment and a BRD4 degrader in a fibroblast‐activation‐protein‐responsive compartment; localized degradation of both targets was associated with improved lung architecture, respiratory function, collagen burden and fibrotic transcriptional programmes in bleomycin‐induced pulmonary fibrosis in mice [74]. This is substantive preclinical proof‐of‐concept, but it does not isolate whether the therapeutic advantage arose from STING degradation itself, spatially restricted delivery, concurrent BRD4 degradation or their combination. This study also does not establish human efficacy, and the available evidence does not justify claims of superior catalytic efficiency, longer functional suppression, lower dosing frequency or proteome‐wide selectivity relative to conventional inhibitors. Accordingly, current evidence favours small‐molecule inhibition as the more extensively tested STING‐directed senomorphic approach, while STING degradation remains an emerging modality whose putative advantages require direct head‐to‐head pharmacological validation. Whether partial or readily reversible pathway suppression would better preserve protective innate immunity than prolonged protein depletion remains an important therapeutic question rather than an established advantage.

### 6.2 Enhancing Mitophagic Flux

Restoring mitochondrial quality control is conceptually upstream because it can remove damaged mitochondria before their contents become persistent innate immune stimuli. Basal mitophagy appears causally relevant to cellular aging: suppressing PINK1/Parkin/p62‐associated basal mitophagy induced aging phenotypes in human cells, whereas mitophagy reactivation was required for the antisenescence effects of NAD+ precursors and rapamycin in the same system [75]. In aged mice and primary fibroblasts from older human donors, cytosolic mtDNA and cGAS–STING‐associated inflammatory signalling increased; urolithin A attenuated pathway activation and neurological decline in old mice, indicating that mitophagy can limit cytosolic mtDNA‐dependent innate immune activation during aging [53]. In APP/PS1 mice, nicotinamide riboside increased brain NAD+, induced mitophagy, reduced ectopic cytoplasmic DNA and cGAS–STING signalling and attenuated neuroinflammation and cellular senescence while improving synaptic and cognitive measures [72]. In senescent muscle cells, NMN/NAD+ restoration promoted mitochondrial repair and mitophagy and reduced mtDNA leakage, cGAS–STING activation and senescence‐associated phenotypes [76]. These studies provide unusually direct support for an upstream mitochondrial quality‐control strategy, although translational development remains predominantly preclinical.

Urolithin A has a broader mitophagy‐specific evidence base than the generic label ‘mitochondrial enhancer’ implies. In UVA‐stressed human dermal fibroblasts, urolithin A attenuated senescence and SASP features while activating a SIRT3–FOXO3–PINK1–Parkin‐associated mitophagy programme. In oxidative‐stress‐exposed auditory cells and cochlear explants, its antisenescence effects were lost when Parkin or BNIP3 signalling was disrupted [77, 78]. Additional studies in *Caenorhabditis elegans* and mammalian cells linked urolithin A‐induced mitophagy to calcium‐dependent organelle communication and showed that calcium chelation abolished the mitophagic response and associated healthspan phenotypes [79]. Evidence in humans is less definitive. In a randomized, placebo‐controlled study, 50 healthy middle‐aged adults received 1000 mg/day of urolithin A for four weeks; the study reported shifts toward naïve‐like CD8 T‐cell phenotypes, increased fatty‐acid oxidation, changes related to mitochondrial biogenesis and innate immune‐cell profiles and improvement in selected immune functions [80]. These findings demonstrate human pharmacodynamic activity but not reversal of cellular senescence, prevention of mtDNA leakage or disease‐modifying benefit. Mitophagy enhancement is therefore best viewed as a mitochondrial‐resilience strategy with credible upstream senomorphic potential. Only selected models have demonstrated the complete causal sequence from enhanced mitochondrial clearance to reduced cytosolic mtDNA and lower cGAS–STING‐driven inflammation.

### 6.3 Advanced Delivery Systems

Targeted delivery addresses a central weakness of systemic innate‐immune modulation: a drug can be mechanistically specific for STING yet remain biologically nonselective across cell states and tissues. The strongest senescent‐cell‐directed proof‐of‐concept in the available literature exploited elevated lysosomal β‐galactosidase activity to release galacto‐oligosaccharide‐encapsulated payloads preferentially in senescent cells; in mice, this strategy increased delivery to senescent cells, improved treatment effects in chemotherapy‐induced senescence and pulmonary fibrosis models and reduced toxic effects of the encapsulated cytotoxic drugs [81]. A different active‐targeting strategy used VCAM‐1‐antibody‐decorated nanocarriers, which produced greater fluorescence in induced and replicative senescent endothelial cells than in healthy endothelial cells, with uptake depending on antibody concentration and VCAM‐1 accessibility [82]. These systems demonstrate differential cell‐state‐associated delivery in defined models, but neither establishes a universal senescence antigen.

Other platforms achieve disease‐compartment or lineage targeting rather than true senescent‐cell specificity. In osteoarthritis models, intra‐articular LNP‐mediated FAP siRNA reduced FAP, NF‐κB‐associated SASP signalling, senescent‐cell burden and cartilage destruction, while a separate LNP platform delivered Zdhhc11 mRNA to chondrocytes in vivo and improved cartilage‐related phenotypes [83, 84]. A macrophage‐directed pCQ/SOD nanoparticle reduced BAX‐dependent mitochondrial outer‐membrane permeabilization, mtDNA release and SASP secretion and improved trauma‐ and aging‐associated osteoarthritis in mice [85]. In age‐related tendinopathy, a ROS‐responsive hydrogel carrying selenium nanocatalysts and H‐151 provided local pathway modulation associated with reduced mtDNA leakage/cGAMP signalling and preservation of tendon‐derived stem‐cell properties [86]. Finally, a collaborative delivery system for intervertebral‐disc degeneration combined sustained IL‐37 release with PLGA nanoparticle delivery of itaconate to remodel SASP‐positive nucleus pulposus cells and inflammatory macrophages [87]. Together, these studies demonstrate the feasibility of spatial restriction and cell‐compartment targeting, but none provides comparative pharmacokinetic evidence that systemic exposure to STING inhibitors is reduced. Delivery technology therefore narrows exposure without ensuring senescent‐cell specificity; its selectivity depends on the biological specificity of the chosen trigger, antigen, lineage marker or diseased microenvironment.

### 6.4 The STING Paradox (Safety & Oncogenesis)

The central safety problem for chronic STING‐directed senomorphics is that the same pathway implicated in sterile age‐associated inflammation also contributes to protective immunity. Genetic destabilization of STING through loss of the deubiquitinase OTUD5 impaired type I interferon signalling and increased susceptibility to HSV‐1 infection and melanoma development in conditional knockout mice, while STING mutants defective in TBK1 recruitment lost antiviral resistance and antitumour responses in a separate mouse study [88, 89]. In cancer therapy, pharmacologically induced micronuclei generated cooperating dsRNA/MAVS and dsDNA/cGAS‐STING signals that increased antigen‐presentation programmes and, with pulsed MPS1 inhibition, promoted cytotoxic‐lymphocyte‐mediated antitumour immunity in vivo [90]. These results raise a concrete concern that indiscriminate or prolonged pathway suppression could weaken beneficial immune functions, but they do not demonstrate that clinically used STING inhibitors cause viral susceptibility or cancer in humans.

The opposite side of the paradox is equally important: STING activity is not uniformly antitumour. In a breast‐cancer model, an EZH2‐HMGA1‐USP7‐dependent cytoplasmic‐chromatin‐fragment pathway activated cGAS–STING‐associated inflammation and promoted metastasis in vivo, demonstrating that tumour context and chronic inflammatory signalling can redirect the pathway toward disease progression [91]. Consequently, the relevant therapeutic variable is not simply activation versus inhibition but biological context, cellular compartment, duration and magnitude of signalling. Preclinical delivery studies show that regional restriction is technically achievable: local ROS‐responsive H‐151 delivery in tendon and inhaled lesion‐responsive STING degradation in fibrotic lung both constrained intervention to diseased compartments in animal models [74, 86]. They do not yet prove that localization preserves antiviral or antitumour immunity better than systemic treatment. Intermittent dosing, partial pathway inhibition, local administration, senescent‐cell‐directed delivery and deliberately reversible suppression therefore remain rational risk‐management concepts rather than validated solutions within the available evidence. The clinical success of STING‐directed senomorphics will depend not simply on whether the pathway can be inhibited, but on controlling where, when, how strongly and for how long it is suppressed. The mechanisms, evidence stages and translational limitations of emerging strategies targeting the mitochondrial–cGAS–STING axis are summarized in Table 3.

**TABLE 3:** Emerging therapeutic strategies targeting the mitochondrial damage–mtDNA–cGAS–STING–SASP axis.

Strategy	Representative interventions	Evidence stage	Main limitation	Representative references
STING inhibition	H‐151; C‐176	Cellular, animal and human ex vivo	Possible suppression of protective immunity	[62, 70, 86, 88, 89]
STING degradation	STING‐targeting PROTAC nanococktail	Animal proof‐of‐concept	No direct comparison with STING inhibitors	[74]
Mitophagy enhancement	Urolithin A; nicotinamide riboside; NMN/NAD^+^ restoration	Cellular and animal; limited human evidence	Clinical senescence reversal remains unproven	[72, 76, 80]
mtDNA‐release inhibition	MOMP suppression; VBIT‐4; pCQ/SOD nanoparticles	Cellular and animal	Release mechanisms vary by biological context	[33, 85]
Senescent‐cell‐directed delivery	Galacto‐encapsulated drugs; VCAM‐1 nanocarriers	Cellular and animal	No universal senescence‐specific target	[81, 82]
Local or tissue‐targeted delivery	H‐151 hydrogel; inhaled PROTAC; intra‐articular LNPs	Animal	Pharmacokinetic and safety advantages remain unconfirmed	[83, 84, 87]

*Note:* Most approaches are senomorphic, because they suppress inflammatory features of senescence without demonstrating selective senescent‐cell elimination.

Abbreviations: LNP, lipid nanoparticle; MOMP, mitochondrial outer‐membrane permeabilization; NMN, nicotinamide mononucleotide; PROTAC, proteolysis‐targeting chimera; SASP, senescence‐associated secretory phenotype.

## 7 Clinical Translation and Future Directions

The central translational question is whether mitochondrial‐stress biology can be measured, attributed specifically to senescence rather than competing injuries and safely modulated in patients. Pathway‐proximal signals are detectable in humans: acute Kawasaki disease was associated with increased serum mtDNA and 2′3′‐cGAMP, whereas a recent systemic lupus erythematosus (SLE) study reported cGAS activation and elevated plasma cGAMP and proposed plasma cGAMP as a response biomarker. That study also relied on patient‐cell and animal experiments [92, 93]. These findings establish target measurability, but not senescence‐specific target validation or therapeutic efficacy in humans. Translation requires evidence that biomarkers identify the relevant biology, drug exposure modifies that biology in patients and pathway modulation improves clinically meaningful outcomes without unacceptable disruption of physiological innate immunity. To clearly distinguish mechanistic and translational evidence, the current level of support for the mitochondrial–mtDNA–cGAS–STING axis is summarized according to experimental stage in Table 4. Figure 5 summarizes the therapeutic balance and staged validation pathway required for clinical translation.

**TABLE 4:** Current level of evidence for targeting the mitochondrial–mtDNA–cGAS–STING axis.

Evidence level	Current evidence	Main interpretation	Representative references
Cellular studies	Mechanistic studies demonstrate links among mitochondrial dysfunction, mtDNA release, cGAS–STING activation and SASP‐associated responses	Strong mechanistic support in selected experimental systems	[9, 12, 44]
Animal studies	Genetic and pharmacological interventions have reduced inflammatory, senescence‐associated or tissue‐damage phenotypes in several models	Provides preclinical evidence of therapeutic potential	[39, 55, 57]
Human observational/ex vivo studies	mtDNA, cGAMP, cGAS–STING activity and related inflammatory markers have been detected in human samples and patient‐derived cells	Supports pathway relevance and biomarker feasibility, but not clinical efficacy	[46, 92, 93]
Human therapeutic evidence	Direct evidence that targeting this axis improves aging‐ or senescence‐related clinical outcomes is currently lacking	Prospective clinical validation is required	Supported by the overall evidence synthesis in this review

Abbreviations: cGAMP, cyclic GMP–AMP; cGAS, cyclic GMP–AMP synthase; mtDNA, mitochondrial DNA; SASP, senescence‐associated secretory phenotype; STING, stimulator of interferon genes.

**FIGURE 5:**
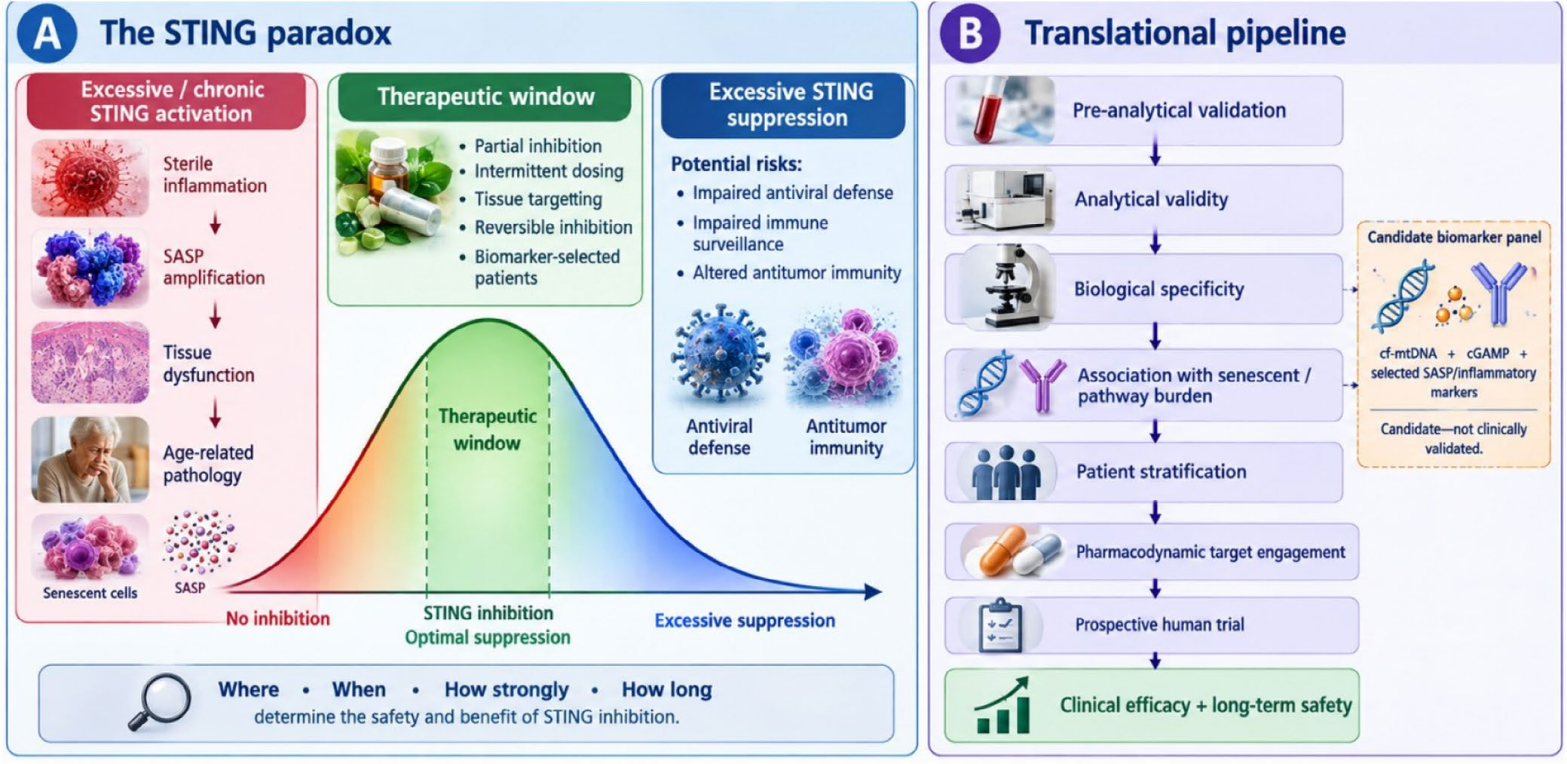
Therapeutic window and translational roadmap for STING‐directed senomorphics. (A) Excessive STING activity promotes SASP‐driven pathology, whereas excessive suppression may compromise protective immunity. (B) Translation requires biomarker validation, patient stratification, pharmacodynamic confirmation and prospective evaluation of efficacy and long‐term safety.

### 7.1 Liquid Biopsies and Biomarkers

Circulating mitochondrial biomarkers are biologically plausible but heterogeneous. In older adults with sarcopenia, plasma cell‐free mtDNA was increased and correlated with IL‐6. A study of Mexican American adults found elevated cell‐free mtDNA in Type 2 diabetes, particularly when cognitive impairment was also present, whereas cellular mtDNA copy number showed different associations [94, 95]. These findings demonstrate disease associations, not that cell‐free mtDNA quantitatively reflects senescent‐cell burden. Acute physiological responses further limit specificity. In healthy men, psychosocial stress and exhaustive exercise increased circulating cfDNA, including cell‐free mtDNA [96]. Acute Kawasaki disease was also associated with increased mtDNA and cGAMP [93]. A single elevated measurement therefore cannot distinguish chronic senescence‐associated mitochondrial stress from acute inflammatory or physiological stress.

Inflammatory and SASP‐associated analytes have similar limitations. Population‐based studies of aging reported age‐associated changes in IL‐6, CXCL8 and CXCL9 and, in a separate nationwide cohort, CXCL10, TNF‐α, IL‐6 and other circulating immune mediators [97, 98]. However, CXCL9, CXCL10 and TNF were also elevated in patients with tuberculosis whose transcriptomic profiles were enriched for cellular‐senescence signatures [99]. An association with aging or senescence‐related biology therefore does not establish specificity for mitochondrial dysfunction, cGAS–STING activity or treatment eligibility. Pathway‐proximal measurements may improve mechanistic interpretation: plasma cGAMP was proposed as a predictor of prasugrel response in SLE, but it has not been validated for senescence‐directed patient selection [92].

Analytical validity is another major barrier. Platelet activation can release extracellular mitochondria and substantially alter measured plasma mtDNA. Direct comparison of plasma‐processing procedures showed marked differences in recovered mtDNA when platelet activation was minimized, identifying platelet‐associated mitochondrial material as a major preanalytical confounder [100]. Studies of cfDNA also show that extraction methods, processing conditions and blood‐cell lysis affect DNA yield and fragment characteristics [101]. Experimental hemolysis increases total plasma cfDNA [102], but current evidence does not establish mtDNA‐specific inflation; hemolysis should therefore be treated as a sample‐quality concern rather than a proven source of increased cell‐free mtDNA. Because studies use different matrices and processing workflows, serum and plasma measurements should not be assumed to be interchangeable. Centrifugation, platelet depletion, storage and freeze–thaw conditions, DNA extraction, assay normalization and the structural form of circulating mtDNA must be standardized before biological cutoffs are credible.

A multimarker strategy is therefore more defensible than reliance on cell‐free mtDNA alone, although its superiority must be demonstrated. A candidate panel could combine a mitochondrial signal, a pathway‐proximal analyte such as cGAMP and selected inflammatory or SASP‐associated factors while distinguishing tissue injury, senescence and pathway activation. Validation should proceed sequentially from analytical validity to biological specificity, association with senescent‐cell burden or pathway activity, longitudinal reproducibility, pharmacodynamic responsiveness and prospective clinical validation. Table 5 summarizes candidate biomarkers, their biological interpretation and the major barriers to clinical validation.

**TABLE 5:** Candidate biomarkers for clinical translation of the mitochondrial–cGAS–STING axis.

Biomarker	Sample	Potential interpretation	Main limitations	Current status	Representative references
Circulating cell‐free mtDNA	Plasma or serum	Mitochondrial damage or extracellular release	Altered by exercise, stress, platelet activation, processing and extraction; not senescence‐specific	Associative biomarker	[93, 94, 95, 100]
Cytosolic mtDNA	Cells or tissue biopsies	Intracellular mtDNA leakage upstream of cGAS	Requires invasive sampling and reliable subcellular fractionation	Mechanistic research biomarker	[46, 47, 53]
2′3′‐cGAMP	Plasma or serum	Pathway‐proximal evidence of cGAS activation	Not specific to senescence; clinical thresholds are unavailable	Candidate pharmacodynamic biomarker	[92, 93]
SASP/inflammatory factors	Plasma or serum	Systemic inflammatory or secretory activity	Strongly influenced by infection and other inflammatory diseases	Nonspecific supportive biomarkers	[97, 98, 99]
Composite panel	Plasma, serum and/or tissue	Combination of mtDNA, cGAMP and selected SASP factors	Requires analytical standardization and prospective validation	Proposed strategy	[92, 93]

*Note:* No individual biomarker currently provides validated, senescence‐specific measurement of mitochondrial–cGAS–STING activity. A composite panel is therefore more defensible, but its superiority remains to be demonstrated.

Abbreviations: cGAMP, cyclic GMP–AMP; cGAS, cyclic GMP–AMP synthase; mtDNA, mitochondrial DNA; SASP, senescence‐associated secretory phenotype; STING, stimulator of interferon genes.

### 7.2 Limitations of the Current Evidence

The current evidence base has several important limitations. First, substantial heterogeneity exists across cell types, tissues, senescence‐inducing stimuli and experimental models, limiting generalization of a single mitochondrial dysfunction–mtDNA–cGAS–STING–SASP pathway across all senescent states. Second, studies frequently assess different mechanistic endpoints, including mitochondrial dysfunction, cytosolic mtDNA, pathway activation, SASP expression or secretion and tissue‐level outcomes, which are related but not interchangeable. Third, much of the causal evidence remains derived from cellular and animal models, while direct evidence of therapeutic efficacy in humans is currently lacking. Biomarker translation is also limited by the absence of standardized and senescence‐specific measures of mitochondrial–cGAS–STING activity. Finally, as this is a narrative review, study selection was based on mechanistic relevance rather than a formal systematic‐review protocol and therefore some degree of selection bias cannot be excluded. These limitations emphasize the need for standardized experimental endpoints, improved biomarker validation and prospective human studies.

### 7.3 Concluding Remarks

The therapeutic problem posed by senescent cells may extend beyond their persistence to mitochondrial–innate immune circuitry that sustains pathological secretory activity. This requires distinguishing senolysis, which eliminates senescent cells; senomorphism, which suppresses or remodels their detrimental phenotype; and mitochondrial–innate immune intervention, which targets upstream mechanisms maintaining inflammatory signalling. These strategies are mechanistically distinct and should not be ranked without direct comparative evidence.

The available evidence does not establish that cGAS‐STING inhibition improves aging or metabolic outcomes in humans. Even the prasugrel study combines human biomarkers with patient‐cell and animal efficacy evidence rather than demonstrating senescence‐directed clinical benefit in treated patients [92]. Moreover, STING biology is context dependent. In EBV‐associated intrahepatic cholangiocarcinoma, experimental pathway activation promoted immunogenic signalling, while patients with this tumour subtype showed higher plasma CXCL10 and IFN‐γ and improved outcomes following anti‐PD‐1 therapy compared with non‐EBV cases [103]. This does not demonstrate that STING inhibition is harmful, but it argues against indiscriminate chronic pathway suppression.

Next‐generation senotherapeutics therefore require patient‐, tissue‐ and disease‐specific target selection, together with pharmacodynamic confirmation that the intended pathway is engaged in the relevant biological compartment. Short‐term SASP suppression cannot be assumed to eliminate senescent cells, and mechanistic importance cannot substitute for long‐term safety or clinical benefit. Clinical development should integrate mechanism‐based target selection with biomarker‐guided stratification, pharmacodynamic confirmation, tissue‐specific biology and rigorous prospective evaluation of safety and efficacy in humans. Mitochondrial dysfunction–cGAS–STING signalling remains a compelling mechanistic framework and therapeutic hypothesis, but its clinical relevance requires biomarker‐defined, pharmacodynamically verified, prospective human validation.

## 8 Conclusion

Recent evidence indicates that mitochondrial dysfunction is a context‐dependent amplifier of senescence‐associated inflammation rather than a universal initiator of cellular senescence. The strongest mechanistic studies link defined mitochondrial lesions—including minority mitochondrial outer‐membrane permeabilization (miMOMP), VDAC1 oligomerization, defective mitophagy and abnormal mtDNA composition—to cytosolic mtDNA exposure, cGAS–STING activation and SASP regulation. These events are distinct experimental endpoints, and cGAS–STING activation alone does not identify mtDNA as the initiating ligand. Depending on the biological context, nuclear DNA, micronuclei, retroelements and mitochondrial RNA can activate overlapping inflammatory pathways.

Preclinical evidence therefore supports STING inhibition primarily as a senomorphic, anti‐inflammatory and tissue‐protective strategy rather than a senolytic intervention. Mitophagy enhancement, inhibition of mtDNA release, targeted protein degradation and precision delivery may provide complementary therapeutic opportunities. However, cGAS and STING are not therapeutically equivalent and excessive pathway inhibition may compromise antiviral and antitumour immunity. No current evidence demonstrates that targeting this axis improves aging‐related outcomes in humans. Clinical translation will require standardized biomarkers, tissue‐ and disease‐specific patient selection, pharmacodynamic confirmation of pathway modulation and prospective evaluation of long‐term efficacy and safety.

## Author Contributions

**Hamza Kamaran Hama:** investigation, data curation, validation, visualization. **Mohammed Ali Hama:** investigation, data curation, formal analysis, software, resources, writing – original draft. **Musa M. Zorab:** conceptualization, methodology, formal analysis, validation, writing – review and editing. **Amirreza Gholami:** methodology, validation, visualization, resources, writing – review and editing. **Ammar Mohammed Al‐Farga:** conceptualization, supervision, validation, resources, writing – review and editing, project administration.

## Funding

The authors have nothing to report.

## Conflicts of Interest

The authors declare no conflicts of interest.

## Data Availability

Data sharing not applicable to this article as no datasets were generated or analysed during the current study.
